# Reference genes selection for quantitative gene expression studies in tea green leafhoppers, *Empoasca onukii* Matsuda

**DOI:** 10.1371/journal.pone.0205182

**Published:** 2018-10-08

**Authors:** Yongchen Yu, Jin Zhang, Chen Huang, Xiangjie Hou, Xiaoling Sun, Bin Xiao

**Affiliations:** 1 Tea Research Institute, Chinese Academy of Agricultural Sciences, Hangzhou, Zhejiang, China; 2 Key Laboratory of Tea Biology and Resources Utilization, Ministry of Agriculture, Hangzhou, Zhejiang, China; 3 College of Horticulture, Northwest A&F University, Yangling, Shaanxi, China; Chinese Academy of Agricultural Sciences Institute of Plant Protection, CHINA

## Abstract

*Empoasca onukii* Matsuda is one of the most devastating pests of the tea plant (*Camellia sinensis*). Still, the presumed expression stability of its reference genes (RGs) has not been analyzed. RGs are essential for accurate and reliable gene expression analysis, so this absence has hampered the study of the insect’s molecular biology. To find candidate RGs for normalizing gene expression data, we cloned ten common housekeeping genes from *E*. *onukii*. Using the ΔCt method, geNorm, NormFinder and BestKeeper, we screened the RGs that were appropriate for quantifying the mRNA transcription of cellular responses under five experimental conditions. We identified the combinations of *α-TUB* and *G6PDH*, *α-TUB* and *UBC*, two RGs (*α-TUB* and *β-TUB1*) or three RGs (*α-TUB*, *RPL13* and *GAPDH*), *AK* and *UBC*, or *RPL13* and α-*TUB* as the best for analyzing gene expression in *E*. *onukii* adults of both sexes in different tissues, nymphs at different developmental stages, nymphs exposed to different temperatures or nymphs exposed to photoperiod stress. Finally, the *E*. *onukii* cysteine proteinase (*Eocyp*) was chosen as the target gene to validate the rationality of the proposed RGs. In conclusion, our study suggests a series of RGs with which to study the gene expression profiles of *E*. *onukii* that have been manipulated (biotically or abiotically) using reverse transcription quantitative polymerase chain reaction. The results offer a solid foundation for further studies of the molecular biology of *E*. *onukii*.

## Introduction

Reverse transcription quantitative polymerase chain reaction (RT-qPCR, hereafter qPCR) is a popular and indispensable technique for quantifying the mRNA transcription of cellular responses triggered by biotic or abiotic manipulations [1]. qPCR offers high-throughput screening, and is known to be fast, sensitive and accurate [2–4]. Every step of qPCR sample preparation and processing—determining the intrinsic variability of RNA, removing impurities during RNA extraction, carrying out reverse transcription and measuring PCR efficiencies—needs to be accurately normalized [5–7]. For now, stably expressed reference genes (RGs) are the best internal controls when results are quantified using the 2^-ΔΔCt^ method or its modified versions [8, 9]. The measurement of internal controls along with target genes helps to compensate for the inevitable experimental variations, such as disparities in the amount of starting material and/or sample loading [10, 11]. Therefore, identifying appropriate RGs for a normalization scalar is an essential prerequisite for developing a qPCR assay. Furthermore, at least two RGs (preferably more) should be employed simultaneously in the normalization process [12–15].

Ideally, RGs should display constitutive and stable expression characteristics across cell lines, tissue types, developmental stages or experimental treatments, and should also be expressed at levels similar to those of target genes [5, 6]. Appropriate RGs are referred to as housekeeping genes. Although *glyceraldehyde-3-phosphate* (*GAPDH*), *18S ribosomal RNA* (*18S rRNA*) and *β-actin* (*ACTB*) have been widely used in gene expression assays of invertebrates [16–19], increasing evidence has demonstrated that these genes have been used without proper validation [12, 20, 21]. Recent experiments have determined the most appropriate RGs for manipulations (biotic and abiotic) of the following species: *Drosophila melanogaster* (Meigen), *Plutella xylostella* (Linnaeus), *Bemisia tabaci* Mediterranean, *Spodoptera litura* (Fabricius), *Sesamia inferens* (Walker), *S*. *exigua* (Hübner), *Bactrocera minax* (Enderlein), *Helicoverpa armigera* Hübner, *Myzus persicae* (Sulzer), *Nilaparvata lugens* (Stål) and *Galeruca daurica* (Joannis) [22–32]. Results from these studies suggest that although RG expression is sometimes constant, it may vary considerably according to experimental treatment or to species. For example, arginine kinase (*AK*) was ranked as the most stable gene when *N*. *lugens* was exposed to temperature-induced stress or examined at different developmental stages, but *AK* was ranked as the most variable gene in the different tissues of the same species [33]. Moreover, *ACTB* in *S*. *litura* was ranked as the second most stable expression gene when insects were treated with insecticide but it was ranked as the most variable one when *S*. *litura* were fed on different foods or when we looked at different tissues [23]. By identifying the proper RGs for a given species under specific experimental conditions, we can avoid missing or overemphasizing potential biological changes in the expression of target genes.

The tea green leafhopper, *Empoasca onukii* Matsuda, one of the most devastating pests of the tea plant (*Camellia sinensis* (L.) O. Kuntze), usually produces ten generations per year in China [34, 35]. Both nymphs and adults of *E*. *onukii* attack the buds, tender leaves and stems of tea plants, causing plant parts to curl and parch [36]. Outbreaks of this hard-to-control insect can cause economic losses of up to 33% due to diminished tea yields [37]. A few molecular studies focusing on species assignment or transcriptome have investigated *E*. *onukii* [36, 38, 39] but little attention has been paid to gene expression analysis or even to the presumed expression stability of RGs. In order to obtain candidate RGs that were appropriate for quantifying the mRNA transcription of cellular responses under five experimental conditions, we cloned ten common RGs with different functions from *E*. *onukii*. Next, using qPCR, we measured ten mRNA transcriptional levels (*ribosomal protein L13* (*RPL13*), *alpha tubulin* (*α-TUB*), *ubiquitin conjugating enzyme* (*UBC*), *glutathione-S-transferase* (*GST*), *glyceraldehyde-3-phosphate dehydrogenase* (*GAPDH*), *TATA-box binding protein* (*TBP*), *glucose-6-phosphate dehydrogenase* (*G6PDH*), *AK* and two *β*-*tubulins* (*β-TUB1* and *β-TUB2*) in the whole bodies, in tissues from different body parts in male or female adults, in nymphs at different life stages and, finally, in nymphs treated with both temperature-induced stress and photoperiod-induced stress. The results were evaluated by using BestKeeper, geNorm, NormFinder and the ΔCt method to identify the most stably expressed RGs [12, 40–42]; an online tool, RefFinder, was then used to integrate the results to find the most stable. Finally, to demonstrate the importance of stable scalar in the normalization process of *E*. *onukii* gene expression, the *E*. *onukii* cysteine proteinase, *Eocyp*, which is expressed in all tissues and at all stages [43], was chosen. Our results identify a series of RGs that could be used with qPCR to study the gene expression profiles of *E*. *onukii* treated with induced stresses (biotic or abiotic); these will provide a solid foundation for further studies of the molecular biology of *E*. *onukii*.

## Methods and materials

### Experimental insects

Mixed-age *E*. *onuki* adults were collected from a tea plantation at the Tea Research Institute of the Chinese Academy of Agricultural Sciences (N 30°10', E 120°5'), Hangzhou, China. Adults were transported to freshly potted tea shoots in enclosed net cages (75 × 75 × 75 cm) and kept in a controlled climate room that was programmed at 26±2°C, 70±5% r.h., under a photoperiod of 14:10 h (L:D). Female adults laid eggs on the tender stems. After one generation, nymphs at different developmental stages or male and female adults were used for different treatments. All samples were frozen instantaneously with liquid nitrogen and stored in a -80°C refrigerator until use. Three biological replicates of all the treatments were prepared. The treatments are briefly summarized below (Table 1).

**Table 1:** Treatments and results.

No.	Treatments			Number of insects in each treatment	Recommended RGs for each treatment
	Name	Material	Condition		
1	Developmental stages of nymphs	First-instar	Whole body	70	*α-TUB*, *UBC*
		Second-instar	Whole body	50	
		Third-instar	Whole body	30	
		Fourth-instar	Whole body	20	
		Fifth-instar	Whole body	20	
2	Sex	Male adults	Whole body	10	*α-TUB*, *G6PDH*
		Female adults	Whole body	10	
3	Different tissues	Male adults	Head	20	*α-TUB*, *RPL13*,*GAPDH*
			Thorax	20	
			Abdomen	20	
		Female adults	Head	20	*α-TUB*, *β-TUB1*
			Thorax	20	
			Abdomen	20	
4	Temperatures	Fifth-instar	Whole body, 4°C	10	*AK*, *UBC*
			Whole body, 26°C	10	
			36°C	10	
5	Photoperiod	Fourth-instar	Whole body, 0:24h (L:D)	10	*RPL13*, *α-TUB*
			Whole body, 14:10h (L:D)	10	
			Whole body, 24:0h (L:D)	10	

#### Developmental stages of nymphs

Five treatment groups, composed of nymphs from the first through the fifth instars, were established. As some groups had few members, large numbers of nymphs at the same developmental stage (70 first-instar, 50 second-instar, 30 third-instar, 20 fourth-instar and 20 fifth-instar) were pooled separately for RNA extraction. Nymphs were separated by morphological characteristics under the microscope (Olympus SZ61, Beijing, China) and collected on the first day of molt.

#### Sexes

Ten two-day-old virgin adult males and females were collected and used for separate analyses.

#### Tissues

Two-day-old virgin male or female adults were dissected (head, thorax, and abdomen) by micro-forceps in liquid nitrogen under the microscope. Tissue from 20 male or female adults was collected and pooled as one sample.

#### Temperatures

Ten fifth-instar nymphs (newly molted) were pooled in a glass tube (diameter 1.5cm, length 8.2cm) with one fresh tea shoot per sample. Tubes were then exposed for 1h to three temperature gradients in a metal bath: low (4°C), moderate (26°C) and high (36°C). The nymphs were allowed to recover at 26°C for another hour. Three sample pools (ten nymphs each) of each treatment were collected separately for RNA extraction. There was no mortality in response to the treatment.

#### Photoperiods

The stability of candidate RGs was tested in fourth-instar nymphs subjected to three different photoperiod treatments in illuminated incubators (SenXinRGQ-360N, Shanghai, China) that were programmed at 26±2°C, 70±5% r.h.: 0:24 h (L:D), 14:10 h (L:D) and 24:0 h (L:D). Two days later, three sample pools (ten nymphs each) of each treatment were collected separately for RNA extraction. There was no mortality in response to the photoperiod treatment.

### Total RNA isolation and cDNA synthesis

Total RNA was isolated by Promega SV Total RNA Isolation System according to the manufacturer’s protocol. The quantity and quality of extracted RNA were confirmed with NANODROP 2000c (Thermo Scientific, Wilmington, DE, USA). Ratios of A260/280 ranged from 2.0 to 2.2, suggesting a high level of purity among all RNA samples. One μg of RNA was used to synthesize the first-strand complementary DNA fragment using PrimeScript RT Master Mix (perfect real time) (TaKaRa, Tokyo, Japan), according to the manufacturer’s protocol. The standard curves were created with a five-fold dilution series of cDNA as a template for each treatment using a liner regression model. The cDNA of all samples was stored at -20°C.

### Cloning and sequence identification

Ten housekeeping genes (*α-TUB*, *AK*, *GAPDH*, *β-TUB1*, *RPL13*, *GST*, *β-TUB2*, *TBP*, *G6PDH* and *UBC*) were selected as candidate RGs from the transcriptome database of *E*. *onukii*. These genes were cloned and sequenced, then confirmed by BLASTX. Primer premier 5 was used for primer design to clone these genes. Full-length cDNA was amplified by PCR using primers shown in Table 2. Each reaction included 50 ng cDNA, 1 μl of each primer (10 μM) and 2X PrimeSTAR Max Premix (TaKaRa, Tokyo, Japan), and the volume was adjusted with nuclease-free water to 20 μl. The PCR program contained a preliminary step at 95°C for 5min, 40 cycles of denaturation at 95°C for 10 s, an annealing temperature for 6s and 58°C for 1–3 min (depending on the length of mRNA). PCR products were examined by gel electrophoresis, purified using a TaKaRa MiniBEST Agarose Gel DNA Extraction Kit Ver 4.0 (TaKaRa, Tokyo, Japan), connected to the pMD 19-T Vector Cloning Kit (TaKaRa, Tokyo, Japan), following the manufacturer’s protocol, and cloned in *Escherichia coli*; three of the bacteria solutions were then sent to Genscript (Nanjing, China) for sequencing. The obtained sequences were compared to the transcriptomic database to confirm the sequence prediction, and an online tool (https://www.genscript.com/tools/real-time-pcr-tagman-primer-design-tool) was used for qPCR primer design (Table 3).

**Table 2:** Sequence information of the Candidate Reference Genes.

Gene Name	Symbol	Forward Primer	Reverse Primer	Amplicon Size (bp)
***ribosomal protein L13***	*RPL13*	TGAAAAATGGCTCCCAAA	CACAAGACATAACCGTATAAAA	734
***alpha tubulin***	*α-TUB*	TGTTGCGTCACTTCGTCT	AGTCAGTTGCGGAAATAAA	1588
***ubiquitin conjugating enzyme***	*UBC*	ATTGCCTGTATGAAAAAAAA	AGGAGCTGATGTCACTTGTG	845
***TATA-Box binding protein***	*TBP*	AATTAACTTAACCATTTCATTT	ACTAACACGTACACTTACACG	981
***glutathione-S-transferase***	*GST*	TACCCTGGTGAGGGTGTC	TCATGCTTTCTTGGTGAGA	823
***glyceraldehyde-3-phosphate dehydrogenase***	*GAPDH*	ACTTTCCTCTTCGTGCCCTTGAAGT	TTGTGAAAAAAATCATGGGCTC	1191
***glucose-6-phosphate dehydrogenase***	*G6PDH*	AACAGAAGAGACTCTGCAGAT	GAGCGTAATTAAGTTAAGGAA	2373
***beta tubulin-1***	*β-TUB1*	CTTAAAGGAATGTTTACCGATT	TCAGGCAGTTCACTTGTTTC	1588
***arginine kinase***	*AK*	GCCCGACCTGACGCAACCTCGCCGC	TCAGTACCCCAGCTATCTGTTT	1633
***beta tubulin-1***	*β-TUB2*	TTACTGACAAGTTATTGGGCG	TGGGAAACACTATTTTCTAAGATA	1620

**Table 3:** Primers of Candidate Reference Genes in *E*.*onukii* for qPCR.

Symbol	Forward Primer	Reverse Primer	Amplicon size (bp)	Efficiency	R^2^
***RPL13***	CGCGCCATCACTGAAGAGGA	CAGCCTCTGGTCAGCTCGTG	75	104.8	0.9997
***α-TUB***	GTGGTGCCAGGAGGTGACTT	ACCCTCTCCGACGTACCAGT	153	100.2	0.9973
***UBC***	GATGCTGAGGCAGACGGACT	TCCCGAGGTGACGTTTGTCG	133	101.2	0.9976
***TBP***	TGGGCTGCAAACTGGACCTG	AAGATTAGCGCCGTCGTCCG	121	109	0.9963
***GST***	TCGCCGATATCGCTCTTGCC	CGTAGCCGGGCAGAGATGAC	120	95.7	0.9992
***GAPDH***	GCTCCTCTCGCCAAGGTCAT	GCGGCGGGAATGATGTTCTG	158	109.5	0.998
***G6PDH***	GAAGGCCCACAGGGTGTTCT	GAGGACATGCAGGTGTTGCG	87	105.2	0.996
***β-TUB1***	ATCCTGCCTCGGAAGATGGC	TCCCGCGTCTCCACTTCTTC	184	101.3	0.9984
***AK***	CAAGCTCGAGGAGGTCGCTG	CTCGGTGAGTCCCATTCGCC	121	99.34	0.9927
***β-TUB2***	ACACCCACCTACGGAGACCT	TCGTGCTGTCAGTGGAGCAA	174	106.9	0.9976
***Eocyp***	CGTCGGCAGATGTGTTCCAA	TGGTCCCAAGCAGAGTCGAT	142	95.6	0.9927

### qPCR analysis

qPCR reactions were performed using a fluorescent quantitative system (LightCycler 480 Ⅱ) based on Synergy Brands. The 10μl reaction system contained 5 μl FastStart Essential DNA Green Master, 0.5 μl of forward and reverse primers (10 μM) and 25 ng of first-strand complementary DNA. The PCR program for all the genes included an initial denaturation step for 10 min at 95°C, followed by 45 cycles of 15s at 95°C, 15s at 60°C and 12s at 72°C. Finally, a melting curve analysis from 60°C to 95°C was performed to confirm the specificity of PCR products. Expression levels of these genes were determined by the number of cycles needed for the amplification to reach a fixed threshold in the exponential phase of the PCR reaction. Triplicates were carried out for each sample.

### Validation of selected reference genes

To demonstrate the importance of proper RGs in the analysis of gene expression profiles, *Eocyp* (Genbank accession number: MH036890) was selected as the target gene. Three different normalization factors (NFs) were calculated based on the geometric mean of the genes with the lowest and the highest geometric mean values as determined by RefFinder, and a single RG with the lowest or the highest geometric mean value. Raw Ct values were transformed to relative quantities using the ΔΔCt method.

### Data analysis

The stability of the candidate RGs was evaluated by BestKeeper, geNorm, NormFinder, the ΔCt method and the online tool RefFinder (http://150.216.56.64/referencegene.php?type=reference). The ΔCt method, geNorm and NormFinder rely on transformed Ct values of (1+E) –^ΔCt^, while original Ct values were used in BestKeeper and RefFinder. All these methods can recommend the most stable RGs; geNorm can also compare the pair-wise variation (V) of one gene with others. The value of V_n_/_n+1_ indicates the pair-wise variation between two sequential NF and the optimal number of RGs required for accurate normalization; these two variables show the optimal number of RGs. Comparisons among more than two samples were analyzed using one-way ANOVA (Tukey’s test); those between two samples were analyzed using Student’s *t*-test.

## Results

### Identification, amplification and PCR efficiency for qPCR of *E*. *onukii* reference genes

Screening by PCR using primers (Table 2) yielded a single amplicon of the expected size for each RG. Each amplicon was cloned, sequenced and annotated in the GenBank database as follows: *α-TUB*, *AK*, *GAPDH*, *β-TUB1*, *RPL13*, *GST*, *β-TUB2*, *G6PDH* and *UBC*. For each pair of primers, the single normalized melting peak suggested that each pair of the primers amplified a unique product. The amplification efficiency (*E*) values for the 10 RGs ranged from 95.6 to 109.5%, with *R*^*2*^ values from 0.9927 to 0.9997 (Table 3), making all assays suitable for quantitative analysis.

### Expression profiles of Candidate Reference Genes

**Fig 1:**
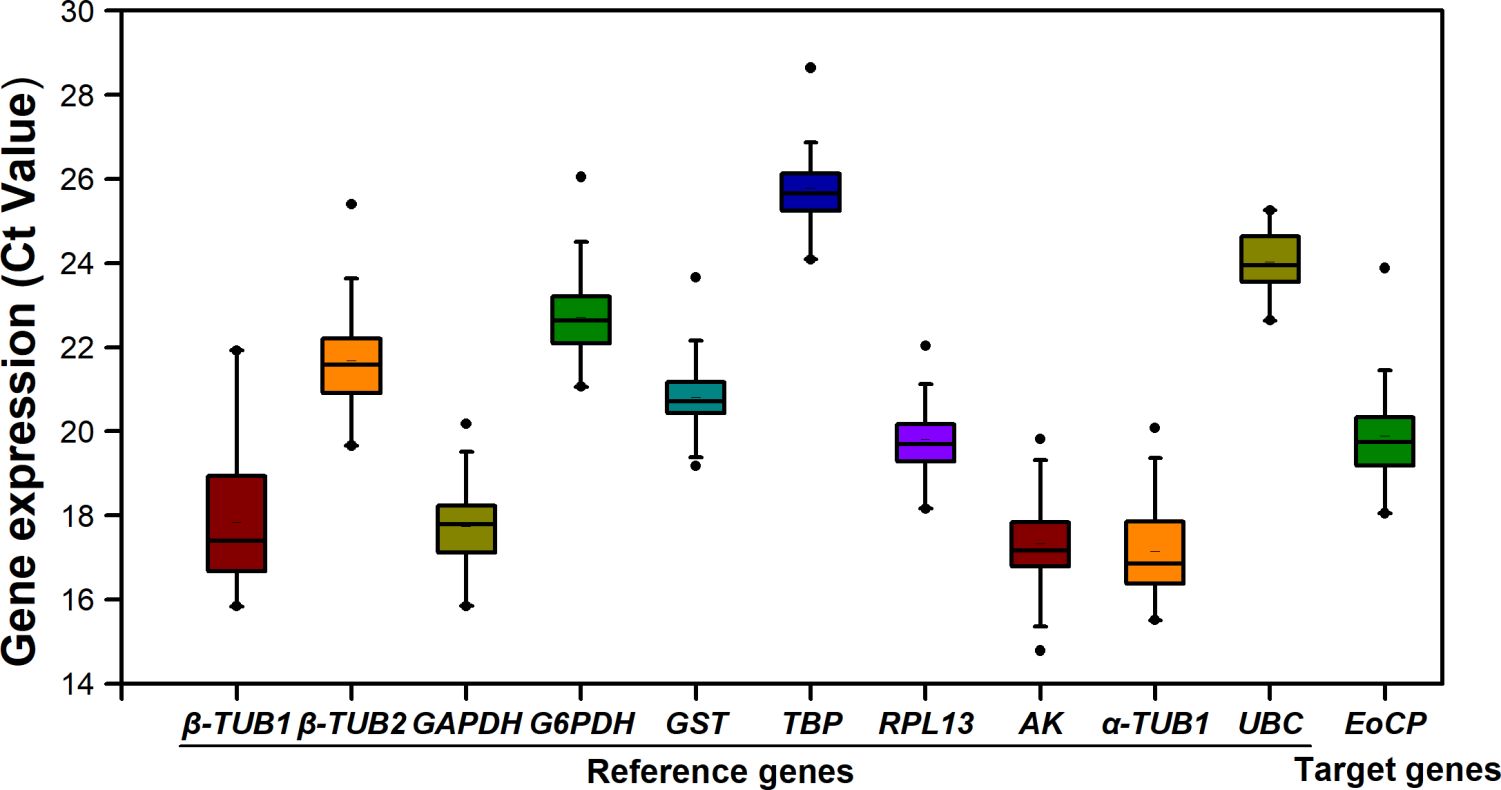
Expression profiles of Candidate Reference Genes in *E*. *onukii*. The expression level of RGs in all samples is documented in terms of the cycle threshold number (Ct value). The data are expressed as box-whisker plots; the short bar in the box refers to Ct mean value; the box represents the 25th–75th percentiles; the median is indicated by a bar across the box; the whiskers on each box represent the distribution of the Ct values; and the dark spots refer to extreme outliers.

As shown in Fig 1, raw Ct values of all candidate RGs ranged from 14.78 (*AK*) to 30.75 (*TBP*). *α-TUB* (17.31), *AK* (17.52), *GAPDH* (17.92) and *β-TUB1* (18.08) were the most abundant transcripts, reaching the threshold fluorescence peak after 18 cycles. *UBC* (24.12) and *TBP* (25.95) were expressed at the lowest levels.

### Developmental stages of Nymphs

**Fig 2:**
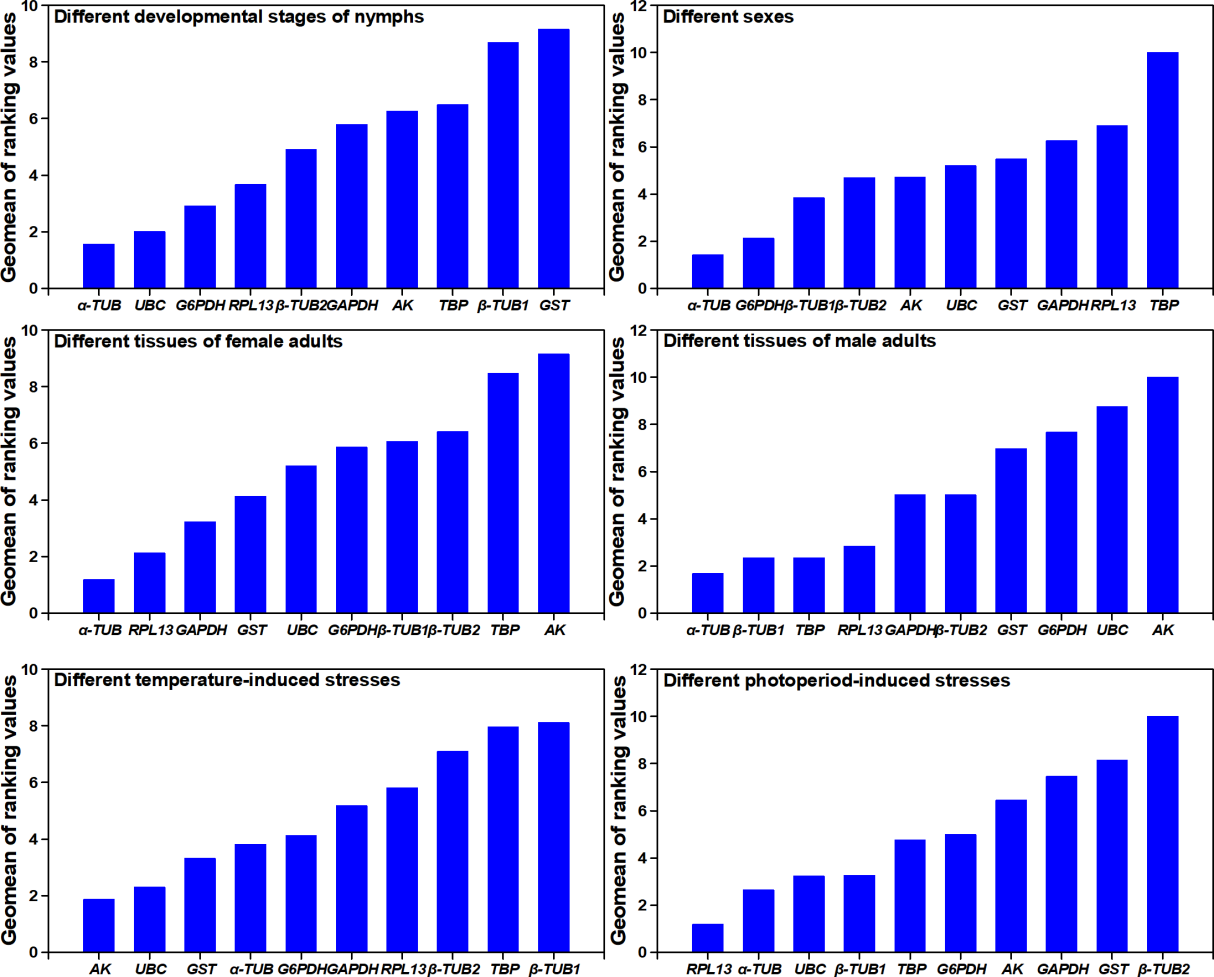
Expression stability of Candidate Reference Genes in *E*. *onukii*. The stability of RG expression was measured by RefFinder. A lower geometric mean value represents more stable expression.

**Fig 3:**
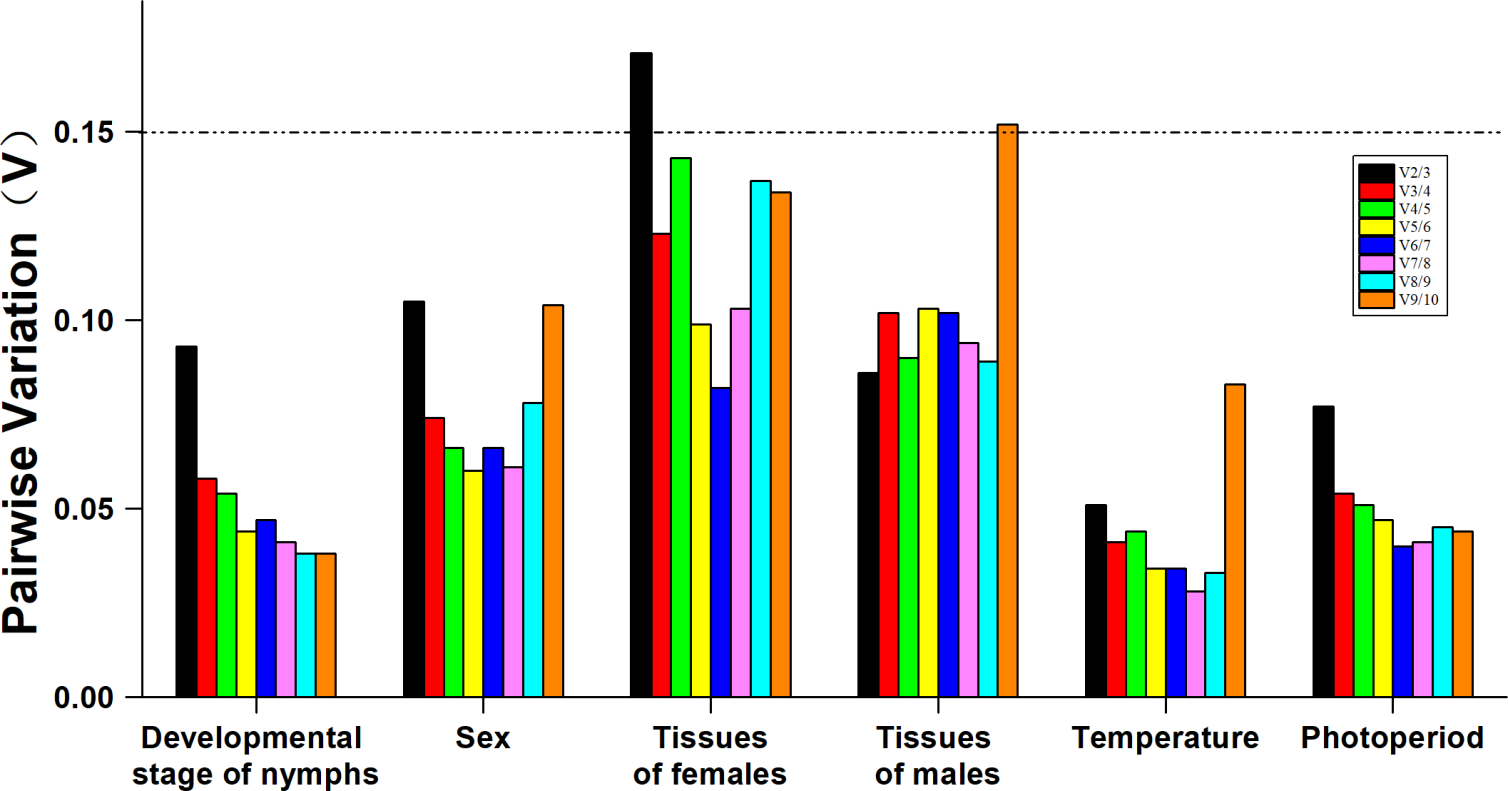
Optimal number of reference genes for the normalization of *E*. *onukii* under different experimental manipulations. The pairwise variation (Vn/n + 1) was analyzed by geNorm software to determine the optimal number of RGs included in the qPCR analysis. Values less than 0.15 indicate that another RG will not significantly improve normalization.

The gene expression stability of ten candidate RGs from nymphs at different developmental stages was analyzed using geNorm, the ΔCt method, BestKeeper and NormFinder. Results showed that the gene stability ranking as analyzed by geNorm differed from the ranking as analyzed by the other three methods (Table 4). For example, approaches using the ΔCt method, BestKeeper or NormFinder across the developmental stages of all nymphs identified *α-TUB* and *UBC* as the most stable RGs, whereas the geNorm approach identified the most stable genes across all developmental stages of all nymphs as *RPL13* and *G6PDH*. However, all four methods all identified *GST* as the most variable. According to results using RefFinder, the ranking from the most to the least stable RG was as follows: *α-TUB* >*UBC* >*G6PDH* >*RPL13* >*β-TUB2* >*GAPDH* >*AK* >*TBP* >*β-TUB1* >*GST* (Fig **2**A). With GeNorm (Fig 3), all pairwise variation (Vn/n+1) was below 0.15 (the recommended cut-off), indicating that the inclusion of an additional RG was unnecessary (Fig 3). Based on the ranking of the RGs by RefFinder, *α-TUB* and *UBC* were identified as the best combination for the developmental stages of nymph *E*. *onukii*.

**Table 4:** Ranking of reference genes expression under different experimental treatments.

Experimental Conditions	Reference Gene	geNorm		NormFinder		BestKeeper			ΔCt	
		Stability	Rank	Stability	Rank	Standard Deviation	Rank	*r*	Standard Deviation	Rank
**Developmental stages of nymphs**	*RPL13*	0.186	1	0.275	6	0.308	5	0.737	0.371	6
	*α-TUB*	0.256	3	0.13	1	0.266	2	0.902	0.302	1
	*UBC*	0.266	4	0.144	2	0.234	1	0.859	0.308	2
	*TBP*	0.351	9	0.307	7	0.276	4	0.571	0.395	7
	*GST*	0.369	10	0.368	10	0.357	7	0.579	0.444	10
	*GAPDH*	0.284	5	0.241	5	0.375	9	0.909	0.354	5
	*G6PDH*	0.186	1	0.236	4	0.35	6	0.84	0.349	3
	*β-TUB1*	0.319	7	0.326	9	0.381	10	0.754	0.41	9
	*AK*	0.336	8	0.315	8	0.272	3	0.59	0.407	8
	*β-TUB2*	0.295	6	0.227	3	0.366	8	0.899	0.35	4
**Sexes**	*RPL13*	0.368	5	0.335	6	0.833	9	0.96	0.57	7
	*α-TUB*	0.22	1	0.11	1	0.533	4	0.989	0.457	1
	*UBC*	0.506	9	0.79	9	0.166	1	0.073	0.84	9
	*TBP*	0.618	10	1.033	10	1.468	10	0.987	1.063	10
	*GST*	0.343	4	0.313	5	0.718	6	0.92	0.56	6
	*GAPDH*	0.439	8	0.486	8	0.366	3	0.78	0.619	8
	*G6PDH*	0.22	1	0.115	2	0.578	5	0.966	0.473	2
	*β-TUB1*	0.296	3	0.23	3	0.825	8	0.991	0.511	3
	*AK*	0.408	7	0.375	7	0.323	2	0.902	0.559	5
	*β-TUB2*	0.319	4	0.232	4	0.722	7	0.974	0.525	4
**Tissues of female adults**	*RPL13*	0.287	1	0.315	2	1.5	5	0.982	0.78	2
	*α-TUB*	0.287	1	0.239	1	1.347	2	0.996	0.752	1
	*UBC*	0.848	9	1.234	9	0.616	1	0.954	1.356	9
	*TBP*	0.722	8	0.927	8	2.157	10	0.964	1.107	8
	*GST*	0.503	4	0.363	4	1.71	6	0.985	0.813	3
	*GAPDH*	0.45	3	0.357	3	1.409	3	0.981	0.814	4
	*G6PDH*	0.656	7	0.561	6	1.415	4	0.951	0.881	7
	*β-TUB1*	0.613	5	0.558	5	1.962	9	0.995	0.867	6
	*AK*	0.967	10	1.323	10	1.755	7	0.888	1.445	10
	*β-TUB2*	0.637	6	0.565	7	1.874	8	0.98	0.86	5
**Tissues of male adults**	*RPL13*	0.246	1	0.338	4	0.441	4	0.616	0.675	4
	*α-TUB*	0.352	4	0.154	2	0.195	1	0.685	0.647	1
	*UBC*	0.681	9	0.887	9	0.794	8	0.234	1.011	9
	*TBP*	0.246	1	0.276	3	0.444	5	0.661	0.648	2
	*GST*	0.625	8	0.603	7	0.452	6	0.361	0.865	7
	*GAPDH*	0.566	7	0.462	5	0.377	3	0.709	0.794	6
	*G6PDH*	0.487	6	0.818	8	0.854	9	0.631	0.949	8
	*β-TUB1*	0.406	5	0.074	1	0.271	2	0.81	0.672	3
	*AK*	0.853	10	1.507	10	1.038	10	0.001	1.541	10
	*β-TUB2*	0.273	3	0.491	6	0.556	7	0.647	0.729	5
**Temperatures**	*RPL13*	0.26	9	0.246	7	0.316	2	0.846	0.377	9
	*α-TUB*	0.155	1	0.192	6	0.465	7	0.952	0.317	5
	*UBC*	0.231	7	0.079	1	0.302	1	0.964	0.31	4
	*TBP*	0.376	10	0.824	10	0.415	4	0.145	0.842	10
	*GST*	0.179	4	0.142	3	0.417	5	951	0.295	2
	*GAPDH*	0.24	8	0.178	5	0.322	3	0.917	0.325	6
	*G6PDH*	0.166	3	0.147	4	0.474	8	0.977	0.296	3
	*β-TUB1*	0.215	6	0.276	9	0.519	10	0.941	0.361	8
	*AK*	0.155	1	0.09	2	0.44	6	0.988	0.29	1
	*β-TUB2*	0.204	5	0.255	8	0.511	9	0.961	0.351	7
**Photoperiods**	*RPL13*	0.188	1	0.084	1	0.183	2	0.911	0.291	1
	*α-TUB*	0.228	3	0.138	2	0.205	4	0.854	0.309	2
	*UBC*	0.24	4	0.156	3	0.193	3	0.765	0.321	3
	*TBP*	0.315	8	0.301	8	0.141	1	0.035	0.394	8
	*GST*	0.345	9	0.426	9	0.264	6	0.001	0.481	9
	*GAPDH*	0.294	7	0.278	7	0.331	9	0.767	0.378	7
	*G6PDH*	0.26	5	0.242	5	0.228	5	0.663	0.357	5
	*β-TUB1*	0.188	1	0.237	4	0.278	7	0.72	0.354	4
	*AK*	0.281	6	0.25	6	0.282	8	0.683	0.36	6
	*β-TUB2*	0.374	10	0.436	10	0.438	10	0.923	0.49	10

### Sexes

BestKeeper, geNorm, ΔCt method and NormFinder identified *α-TUB* and *G6PDH* as the most stable RGs, and *TBP* as the least stable RG (Table 4). According to results from RefFinder, the ranking from the most to the least stable was as follows: *α-TUB* >*G6PDH* >*β-TUB1* >*β-TUB2* >*AK* >*UBC* >*GST* > *GAPDH* >*RPL13* >*TBP* (Fig 2B). Based on results of geNorm, two RGs were suggested. According to RefFinder, *α-TUB* and *G6PDH* were chosen as the best combination for normalizing the expression of *E*. *onukii* adults in different sexes.

### Tissues

Analyses of the ΔCt method, NormFinder and geNorm divided the ten RGs into two groups, each with different tissues of female *E*. *onukii*: the group of more stably expressed genes contained *α-TUB*, *RPL13* and *GAPDH*; the group of less stable expressed genes contained *AK*, *UBC* and *TBP* (Table 4). The BestKeeper analysis revealed that *UBC*, *α-TUB* and *GAPDH* are the most stably expressed genes. According to the results from RefFinder, the stability ranking from the most to the least was as follows: *α-TUB* >*RPL13* >*GAPDH* >*GST* >*UBC* >*G6PDH* >*β-TUB1* >*β-TUB2* >*TBP* >*AK* (Fig 2C). Meanwhile, the most stable RGs in different tissues of male adults were as follows: BestKeeper and the ΔCt method identified *α-TUB*, NormFinder identified *β-TUB1*, and geNorm identified *TBP*. However, all four methods identified *AK* as the most unstable gene (Table 4). Using geNorm and RefFinder in different tissues of female and male adults of *E*. *onukii*, normalization required two (*α-TUB* and *β-TUB1*) and three (*α-TUB*, *RPL13* and *GAPDH*) RGs (Fig 3).

### Temperatures

The ΔCt method and geNorm ranked *AK* as the most stably expressed genes in nymphs exposed to different temperatures, whereas BestKeeper and NormFinder ranked *UBC* as the most stable RG. All analysis programs, except BestKeeper, ranked *TBP* as the most variable gene. RefFinder ranked the genes from the most to the least stable as follows: *AK* >*UBC* >*GST* >*α-TUB* >*G6PDH* >*GAPDH* >*RPL13* >*β-TUB2* >*TBP* >*β-TUB1* (Fig 2E). Furthermore, geNorm and RefFinder analysis revealed that all the V values were below 0.15, which means *AK* and *UBC* are the best RG combination for gene expression analysis when *E*. *onukii* nymphs were exposed to different temperatures (Fig 3).

### Photoperiods

The ΔCt method, NormFinder and geNorm ranked *RPL13*, *α-TUB*, *UBC* and *β-TUB1* as the four most stably expressed genes, and ranked *β-TUB2*, *GST* and *TBP* as the three most variably expressed genes, when nymphs were exposed to different photoperiods. BestKeeper also ranked *RPL13*, *α-TUB* and *UBC* as more stable genes than others, except *TBP*. RefFinder ranked the genes from the most to the least stable: *RPL13* >*α-TUB* >*UBC* >*β-TUB1* >*TBP* >*G6PDH* >*AK* >*GAPDH* >*GST* >*β-TUB2* (Fig 2F). Analysis by geNorm also revealed that all V values were below 0.15 (Fig 3). Thus, the RGs recommended for the nymphs of *E*. *onukii* exposed to photoperiod stress are *RPL13* and *α-TUB*.

### Validation of proposed reference genes

**Fig 4:**
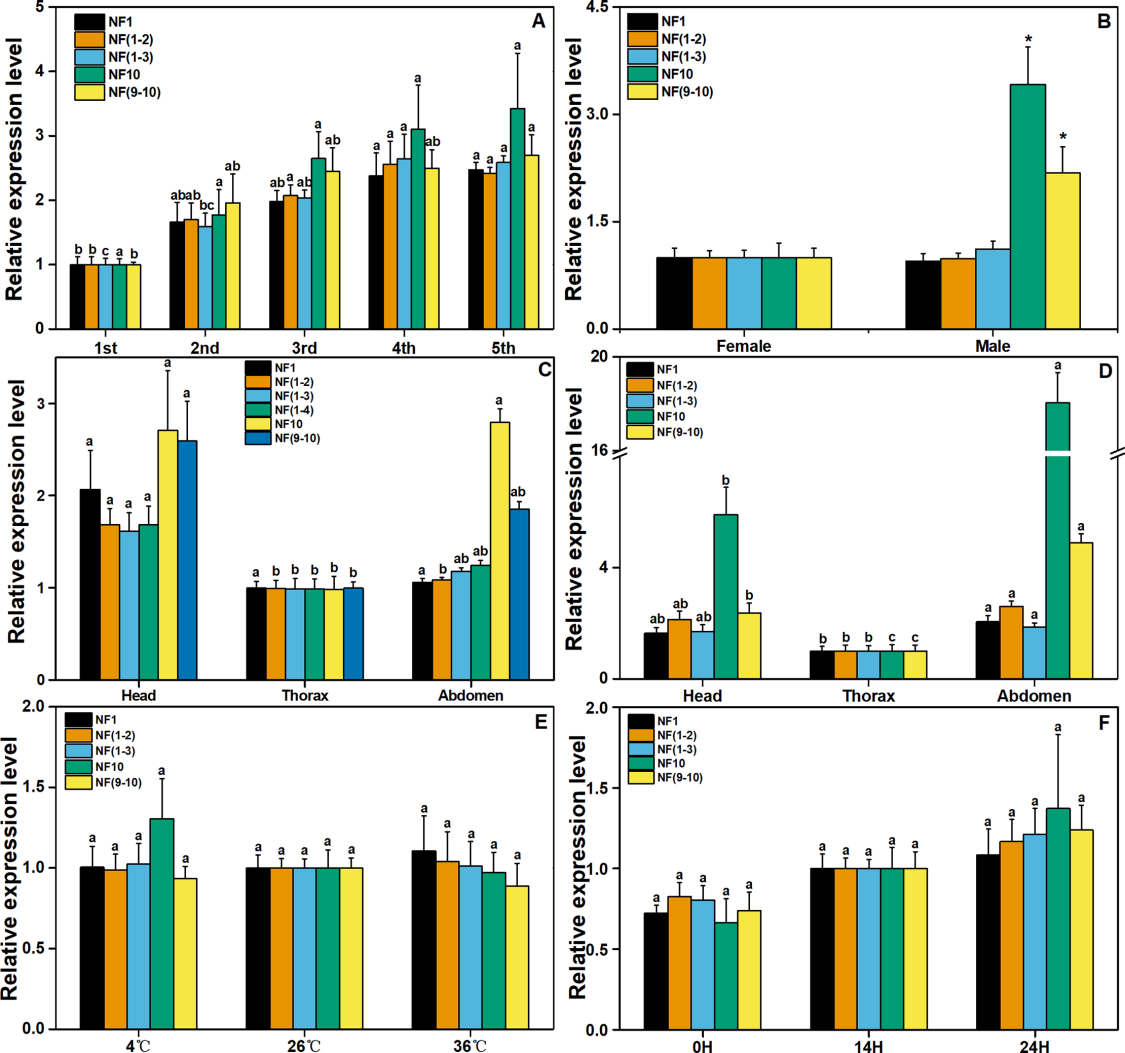
Validation of the gene stability measure. Expression profiles of *Eocyp* under different experimental conditions using different RGs. A. Nymphs at different developmental stages; B. Sex; C. Different tissues in female *E*. *onukii*; D. Different tissues in male *E*. *onukii*; E. Fifth-instar nymphs exposed to different temperatures; F. Fifth-instar nymphs exposed to different photoperiod. Data are means±SE. One-way ANOVA (Tukey’s test) was used to analyze significant difference among treatments (A, C~F); different letters in the same color columns show the statistical difference, *P*<0.05. Two samples were compared using Student’s *t*-test (B); *, *P*<0.05.

*Eocyp* was chosen as the target gene to validate the rationality of the proposed RGs. The expression level of *Eocyp* in the third instar was significantly higher than that in the first instar when normalized with the combination of *α-TUB* and *UBC* (NF 1–2, *F* = 7.997, *P* = 0.004) or *α-TUB*, *UBC* and *G6PDH* (NF 1–3, *F* = 10.498, *P* = 0.001) as RGs, but no significant difference was found when normalized with only one RG (NF1, *F* = 6.537, *P* = 0.007 or NF10, *F* = 3.215, *P* = 0.061) or the combination of the two unstable RGs, *β-TUB1* and *GST*, NF (9–10, *F* = 4.469, *P* = 0.025) (Fig 4A). Analogously, the expression level of *Eocyp* was also not the same when normalized with more than one RG [NF (1–2), *F* = 10.703, *P* = 0.010; NF (1–3), *F* = 5.656, *P* = 0.042; NF (1–4), *F* = 6.706, *P* = 0.030; NF (9–10), *F* = 9.755, *P* = 0.013] compared with compounds normalized with only one RG (NF1, *F* = 5.896, *P* = 0.038 or NF10, *F* = 6.843, *P* = 0.028) in different tissues of female adults. Moreover, when normalized with NF (1–2) (*F* = 7.669, *P* = 0.011), the expression level of *Eocyp* was significantly higher in the head than in the abdomen, but no significant difference was found when *Eocyp* was normalized with other RGs (Fig 4C). When the combination of *α-TUB* and *G6PDH* (NF 1–2, *F* = 0.063, *P* = 0.889) or the combination of *α-TUB*, *G6PDH* and *β-TUB1* (NF 1–3, *F* = 0.058, *P* = 0.469) was used for normalization, similar expression levels of *Eocyp* were observed in female and male adults of *E*. *onukii*. However, when normalized with *TBP* (NF10, *F* = 4.600, *P* = 0.013) or the combination of *PRL13* and *TBP* (NF 9–10, *F* = 1.730, *P* = 0.040), the expression level of *Eocyp* was higher in female adults than that in male adults (Fig 4B). Similar results were observed in different tissues of male adults as well, when normalized with the highest geometric mean value of *AK* (NF10, *F* = 86.973, *P* = 0.000) or the combination of *AK* and *UBC* (NF (9–10), *F* = 42.887, *P* = 0.000), the expression level of *Eocyp* in the head was higher than that in the thorax, whereas it remained the same in the head and thorax when assessed with the most appropriate RG of *α-TUB* (NF1, *F* = 6.696, *P* = 0.017) or the combination of *α-TUB* and *β-TUB1* (NF1-2, *F* = 7.669, *P* = 0.011) (Fig 4D). Moreover, the expression level of *Eocyp* under the two abiotic stresses showed no significant difference when normalized with different individual RGs or combinations of RGs (Fig 4E and 4F).

## Discussion

Accurate normalization is required to minimize errors in qPCR and is a prerequisite for obtaining reliable gene-expression results, especially when the differences are subtle. Many methods to assess the expression stability of RGs have been developed but until now none has been completely reliable. In addition, little attention has been paid to the presumed expression stability of RGs and to gene expression analysis in *E*. *onukii*. As a result, the molecular biology of this pest is not well understood. In the present study, we cloned ten common RGs with different functions from *E*. *onukii* and synthesized four commonly used methods to obtain the most stable RGs; these are needed for quantifying the mRNA transcription of cellular responses in response to five experimental manipulations. The methods—BestKeeper, ΔCt, NormFinder and geNorm—are based on different algorithms [24, 44]. BestKeeper analyzes the stability of the RG individually, whereas the ΔCt method, NormFinder and geNorm analyze pairwise variation between two RGs [45]. In our study, the results from BestKeeper varied somewhat from the results of the other three methods in response to the same treatment. For example, BestKeeper ranked *UBC* as the most stable RG, whereas the other methods identified it as the most unstable in both sexes of *E*. *onukii* (Table4). The negative correlation between *UBC* (r = 0.0073) and the other RGs (Table 4) indicates that the results of BestKeeper were imprecise. A similar phenomenon was found earlier by Pfaffl (2001) [46]. The ranking of RGs by geNorm also differed somewhat from the ranking of other methods when the developmental stages of nymphs were considered (Table 4). Previous results showed that geNorm was not able to evaluate the expression stability of RGs that were co-regulated or expressed similarly; thus, using geNorm presupposed the selection of a pair of RGs [42]. According to Fig 3, if *G6PDH* were excluded, the geNorm ranking of RGs of nymphs at different developmental stages would be changed and the stability of *RPL13* would be correspondingly decreased. However, because the results of NormFinder did not show this trend (S1 Fig), we inferred that geNorm emphasizes the comparisons. Therefore, in order to obtain reliable evaluation results, our study used RefFinder.

In our present study, *α-TUB* exhibited the most stable expression status in response to most biotic manipulations, including the selection of the developmental stages of nymphs and of different tissues in adults of both sexes and in the whole bodies of both sexes (Fig 2). Furthermore, *α-TUB* also exhibited the most stability in different tissues and in the whole bodies of adults of each individual sex and in different tissue from a mixed-sex group of adults (S2–S5 Tables); *G6PDH*, on the other hand, exhibited the most stability across different stages of nymphs and in both sexes (S1 Table). *α-TUB*, which is a cytoskeletal protein and functions in many physiological processes [47], has been ranked as the most stable RG in expression studies under different developmental conditions with *N*. *lugens*, *Liriomyza trifolii*, *B*. *tabaci*, *Tetranychus cinnabarinus*, *Monochamus alternatus* and *Sogatella furcifera* [48–51]. In addition, *α-TUB* was ranked as the most stable RG in different tissues of *C*. *suppressalis*, *Galeruca daurica*, *M*. *alternatus*, *Rhodnius prolixus* and *Phenacoccus solenopsis* [17, 32, 52]. However, *α-TUB* was regarded as a variable RG in response to different developmental conditions of *Apis mellifera* and *Delphacodes kuscheli* [53, 54]. In conclusion, although *α-TUB* was expressed stably in most cases, it should be tested across more species and in response to different treatments before being employed.

Specific RGs may not be completely stable in a species exposed to different experimental conditions or to evaluation by different methods. Sometimes the instability may be due to a gene’s function. *AK*, arginine kinase, the only phosphagen kinase in insects [55], has been found to be the most stable RG in *N*. *lugens* and *C*. *suppressalis* when these were subjected to temperature stresses or insecticides, and also the most stable RG in different larval tissues of *S*. *litura*, and in the labial gland and fat body of *Bombus terrestris* [56, 57]. Our results showed that *AK* was the most stable RG in *E*. *onukii* nymphs exposed to different temperatures (Fig 2E) but it was the most unstable RG in tissues from the head and/or the thorax of both male and female adults (Fig 2B and 2C). *Ribosomal protein 13L* (*RPL13*) encodes a ribosomal protein that is a component of the 60S subunit and, in conjunction with rRNA, constitutes the ribosomal subunits involved in the cellular process of translation [47]. Our results showed that *RPL13* was an optimized RG of *E*. *onukii* in different tissues of female adults and in nymphs that had been exposed to different photoperiods (Fig 2F). Previous results showed that the ribosomal protein gene was found to be the most stable RG in *A*. *mellifera*, *Schistocerca gregaria*, *Tribolium castaneum*, *D*. *melanogaster*, *Bombyx mori*, *C*. *suppressalis* and *B*. *tabaci* under certain experimental conditions [22, 29, 53, 57–60]. *GST* was regarded as the most unstable RG in *E*. *onukii* nymphs at different developmental stages or in nymphs exposed to different photoperiods; however, RefFinder ranked *GST* as the most stable RG when all experimental conditions were considered (S6 Table). In conclusion, the normalization scalars among the candidates correspond to experimental conditions and the best way to select a suitable RG for gene expression analysis is to evaluate it under specific experimental conditions.

Recently, multiple RGs have been used to normalize the expression of target genes more accurately. When multiple RGs were used in a given experiment, the probability of biased normalization was reduced [19, 61–64]. Traditionally, an optimal number of RGs with the least pairwise variation (V) was selected by GeNorm. GeNorm determines the pairwise variations (V_n/n+1_) in NFs (the geometric mean of multiple RGs) using n or n+1 RGs with a threshold value< 0.15 but the threshold value is not totally absolute [12]. In the present study, our results not only confirmed that the most appropriate RGs differed across experimental manipulations but they also proved that the use of multiple RGs in qPCR increased the accuracy and sensibility of gene expression analysis in *E*. *onukii* (Fig 4A–4D). Furthermore, the results demonstrated that when *Eocyp* expression data were normalized with the combination of *α-TUB*, *RPL13* and *GAPDH*, subtle differences among different tissues of female adult *E*. *onukii* were detected but when data were normalized with one RG or with other combinations of RGs, no differences were observed.

In conclusion, using qPCR, we screened a series of RGs to study the gene expression profiles of *E*. *onukii* in response to multiple experimental manipulations. This study provide a solid foundation for further studies of the molecular biology of *E*. *onukii*.

## Supporting information

S1 Table Expression stability of Candidate Reference Genes across different developmental stages of Nymphs and between sexes. (DOCX)

S2 Table Expression stability of Candidate Reference Genes in different tissues and whole bodies of *E*. *onukii* Adult Females. (DOCX)

S3 Table Expression stability of the candidate RGs in different tissues and whole bodies of *E*. *onukii* adult males. (DOCX)

S4 Table Expression stability of the Candidate Reference Genes in different tissues in *E*. *onukii* male and female adults. (DOCX)

S5 Table Expression stability of the Candidate Reference Genes in different tissues and whole body in *E*. *onukii* male and female adults. (DOCX)

S6 Table Expression stability of Candidate Reference Genes under all conditions. (DOCX)

S1 Fig Robustness of geNorm and NormFinder. To assess the robustness of the rankings among the candidate genes, we compared the results obtained using all genes (solid lines), the results obtained when excluding *G6PDH* (dashed lines) or α-*TUB* (dot lines) in response to the developmental stages of nymph *E*. *onukii*. The results described by geNorm use black curves; red curves represent the values described by NormFinder. The top ranked genes are those with the smallest values for each method.
(DOCX)
