# Supplementary material for: Reference genes selection for quantitative gene expression studies in tea green leafhoppers, Empoasca onukii Matsuda
Source: PLoS One. 2018 Oct 8;13(10):e0205182. doi: 10.1371/journal.pone.0205182 (PMC6175517; doi:10.1371/journal.pone.0205182)
Supplement: S1 Table — (DOCX) [file pone.0205182.s001.docx]

**S1 Table. Expression Stability of Candidate Reference Genes across Different Developmental Stages of Nymphs and between Sexes.**

| **Reference gene** | **geNorm** | | **NormFinder** | | **BestKeeper** | | | **ΔC_t_** | | RefFinder | |
| --- | --- | --- | --- | --- | --- | --- | --- | --- | --- | --- | --- |
|  | **Stability** | **Rank** | **Stability** | **Rank** | **Standard deviation** | **Rank** | **r** | **Standard deviation** | **Rank** | **Geomean** | **Rank** |
| *RPL13* | 0.274 | 1 | 0.267 | 3 | 0.669 | 6 | 0.958 | 0.542 | 3 | 2.711 | 2 |
| *α-TUB* | 0.507 | 8 | 0.389 | 5 | 0.931 | 9 | 0.975 | 0.609 | 5 | 6.51 | 8 |
| *UBC* | 0.46 | 6 | 0.499 | 7 | 0.415 | 1 | 0.852 | 0.644 | 7 | 4.141 | 5 |
| *TBP* | 0.55 | 9 | 0.62 | 9 | 0.679 | 7 | 0.794 | 0.753 | 9 | 8.452 | 9 |
| *GST* | 0.48 | 7 | 0.422 | 6 | 0.489 | 3 | 0.856 | 0.616 | 6 | 5.244 | 7 |
| *GAPDH* | 0.443 | 5 | 0.501 | 8 | 0.441 | 2 | 0.828 | 0.646 | 8 | 5.03 | 6 |
| *G6PDH* | 0.274 | 1 | 0.165 | 1 | 0.621 | 5 | 0.968 | 0.5 | 1 | 1.496 | 1 |
| *β-TUB1* | 0.638 | 10 | 0.926 | 10 | 1.303 | 10 | 0.968 | 0.986 | 10 | 10 | 10 |
| *AK* | 0.383 | 4 | 0.332 | 4 | 0.55 | 4 | 0.922 | 0.562 | 4 | 4 | 4 |
| *β-TUB2* | 0.313 | 3 | 0.187 | 2 | 0.724 | 8 | 0.971 | 0.517 | 2 | 3.13 | 3 |
