# Supplementary material for: Reference genes selection for quantitative gene expression studies in tea green leafhoppers, Empoasca onukii Matsuda
Source: PLoS One. 2018 Oct 8;13(10):e0205182. doi: 10.1371/journal.pone.0205182 (PMC6175517; doi:10.1371/journal.pone.0205182)
Supplement: S2 Table — (DOCX) [file pone.0205182.s002.docx]

**S2 Table. Expression Stability of Candidate Reference Genes in Different Tissues and Whole Bodies of *E.* *onukii* Adult Females.**

| **Reference gene** | **geNorm** | | **NormFinder** | | **BestKeeper** | | | **ΔC_t_** | | RefFinder | |
| --- | --- | --- | --- | --- | --- | --- | --- | --- | --- | --- | --- |
|  | **Stability** | **Rank** | **Stability** | **Rank** | **Standard deviation** | **Rank** | **r** | **Standard deviation** | **Rank** | **Geomean** | **Rank** |
| *RPL13* | 0.26 | 1 | 0.351 | 2 | 1.272 | 3 | 0.98 | 0.77 | 2 | 1.861 | 2 |
| *α-TUB* | 0.26 | 1 | 0.332 | 1 | 1.147 | 2 | 0.993 | 0.763 | 1 | 1.189 | 1 |
| *UBC* | 0.927 | 10 | 1.234 | 10 | 0.497 | 1 | 0.928 | 1.346 | 10 | 5.623 | 6 |
| *TBP* | 0.712 | 8 | 0.791 | 8 | 1.87 | 10 | 0.965 | 0.994 | 8 | 8.459 | 9 |
| *GST* | 0.545 | 4 | 0.381 | 4 | 1.524 | 6 | 0.983 | 0.785 | 4 | 4.427 | 4 |
| *GAPDH* | 0.482 | 3 | 0.36 | 3 | 1.304 | 4 | 0.979 | 0.784 | 3 | 3.224 | 3 |
| *G6PDH* | 0.656 | 6 | 0.593 | 6 | 1.447 | 5 | 0.947 | 0.88 | 6 | 5.733 | 7 |
| *β-TUB1* | 0.609 | 5 | 0.469 | 5 | 1.688 | 7 | 0.993 | 0.802 | 5 | 5.439 | 5 |
| *AK* | 0.822 | 9 | 1.122 | 9 | 1.688 | 8 | 0.9 | 1.264 | 9 | 8.739 | 10 |
| *β-TUB2* | 0.672 | 7 | 0.64 | 7 | 1.709 | 9 | 0.979 | 0.883 | 7 | 7.454 | 8 |
