# Supplementary material for: Reference genes selection for quantitative gene expression studies in tea green leafhoppers, Empoasca onukii Matsuda
Source: PLoS One. 2018 Oct 8;13(10):e0205182. doi: 10.1371/journal.pone.0205182 (PMC6175517; doi:10.1371/journal.pone.0205182)
Supplement: S3 Table — (DOCX) [file pone.0205182.s003.docx]

**S3 Table. Expression Stability of the Candidate RGs in Different Tissues and Whole Bodies of *E.* *onukii* Adult Males.**

| **Reference gene** | **geNorm** | | **NormFinder** | | **BestKeeper** | | | **ΔC_t_** | | **RefFinder** | |
| --- | --- | --- | --- | --- | --- | --- | --- | --- | --- | --- | --- |
|  | **Stability** | **Rank** | **Stability** | **Rank** | **Standard deviation** | **Rank** | **r** | **Standard deviation** | **Rank** | **Geomean** | **Rank** |
| *RPL13* | 0.601 | 7 | 0.528 | 6 | 0.447 | 1 | 0.717 | 0.76 | 6 | 3.984 | 6 |
| *α-TUB* | 0.48 | 4 | 0.157 | 1 | 0.583 | 2 | 0.967 | 0.62 | 1 | 1.682 | 1 |
| *UBC* | 0.681 | 9 | 0.801 | 9 | 0.913 | 9 | 0.621 | 0.938 | 9 | 9 | 9 |
| *TBP* | 0.522 | 5 | 0.241 | 2 | 0.673 | 3 | 0.91 | 0.626 | 2 | 2.783 | 2 |
| *GST* | 0.295 | 1 | 0.552 | 7 | 0.711 | 4 | 0.871 | 0.807 | 7 | 3.742 | 5 |
| *GAPDH* | 0.295 | 1 | 0.407 | 4 | 0.711 | 5 | 0.916 | 0.737 | 5 | 3.162 | 3 |
| *G6PDH* | 0.64 | 8 | 0.73 | 8 | 0.744 | 8 | 0.744 | 0.88 | 8 | 8 | 8 |
| *β-TUB1* | 0.446 | 3 | 0.33 | 3 | 0.813 | 6 | 0.979 | 0.705 | 3 | 3.568 | 4 |
| *AK* | 0.822 | 10 | 1.338 | 10 | 1.115 | 10 | 0.515 | 1.385 | 10 | 10 | 10 |
| *β-TUB2* | 0.561 | 6 | 0.492 | 5 | 0.823 | 7 | 0.909 | 0.737 | 4 | 5.384 | 7 |
