# Supplementary material for: Reference genes selection for quantitative gene expression studies in tea green leafhoppers, Empoasca onukii Matsuda
Source: PLoS One. 2018 Oct 8;13(10):e0205182. doi: 10.1371/journal.pone.0205182 (PMC6175517; doi:10.1371/journal.pone.0205182)
Supplement: S4 Table — (DOCX) [file pone.0205182.s004.docx]

**S4 Table. Expression Stability of the Candidate Reference Genes in Different Tissues in *E.* *onukii* Male and Female Adults.**

| **Reference gene** | **geNorm** | | **NormFinder** | | **BestKeeper** | | | **ΔC_t_** | | **RefFinder** | |
| --- | --- | --- | --- | --- | --- | --- | --- | --- | --- | --- | --- |
|  | **Stability** | **Rank** | **Stability** | **Rank** | **Standard deviation** | **Rank** | **r** | **Standard deviation** | **Rank** | **Geomean** | **Rank** |
| *RPL13* | 1.006 | 8 | 1.047 | 8 | 0.833 | 1 | 0.797 | 1.291 | 7 | 4.601 | 4 |
| *α-TUB* | 0.704 | 4 | 0.196 | 1 | 0.889 | 2 | 0.991 | 0.911 | 1 | 1.682 | 1 |
| *UBC* | 0.912 | 7 | 1.022 | 7 | 1.03 | 3 | 0.805 | 1.293 | 8 | 5.856 | 7 |
| *TBP* | 1.062 | 9 | 1.112 | 9 | 1.151 | 4 | 0.795 | 1.342 | 9 | 7.348 | 9 |
| *GST* | 0.533 | 1 | 0.509 | 3 | 1.302 | 5 | 0.97 | 0.991 | 3 | 2.59 | 2 |
| *GAPDH* | 0.533 | 1 | 0.727 | 4 | 1.604 | 8 | 0.96 | 1.082 | 4 | 3.364 | 4 |
| *G6PDH* | 0.803 | 6 | 0.764 | 5 | 1.39 | 7 | 0.923 | 1.109 | 5 | 5.692 | 6 |
| *β-TUB1* | 0.617 | 3 | 0.373 | 2 | 1.333 | 6 | 0.993 | 0.941 | 2 | 2.913 | 3 |
| *AK* | 1.165 | 10 | 1.428 | 10 | 1.698 | 10 | 0.85 | 1.579 | 10 | 10 | 10 |
| *β-TUB2* | 0.769 | 5 | 0.788 | 6 | 1.634 | 9 | 0.971 | 1.114 | 6 | 6.344 | 8 |
