# Supplementary material for: Reference genes selection for quantitative gene expression studies in tea green leafhoppers, Empoasca onukii Matsuda
Source: PLoS One. 2018 Oct 8;13(10):e0205182. doi: 10.1371/journal.pone.0205182 (PMC6175517; doi:10.1371/journal.pone.0205182)
Supplement: S5 Table — (DOCX) [file pone.0205182.s005.docx]

**S5 Table. Expression Stability of the Candidate Reference Genes in Different Tissues and Whole Body in *E.* *onukii* Male and Female Adults.**

| **Reference gene** | **geNorm** | | **NormFinder** | | **BestKeeper** | | | **ΔC_t_** | | RefFinder | |
| --- | --- | --- | --- | --- | --- | --- | --- | --- | --- | --- | --- |
|  | **Stability** | **Rank** | **Stability** | **Rank** | **Standard deviation** | **Rank** | **r** | **Standard deviation** | **Rank** | **Geomean** | **Rank** |
| *RPL13* | 0.933 | 8 | 0.943 | 7 | 0.835 | 1 | 0.792 | 1.185 | 7 | 4.45 | 5 |
| *α-TUB* | 0.665 | 4 | 0.193 | 1 | 0.854 | 2 | 0.988 | 0.844 | 1 | 1.682 | 1 |
| *UBC* | 0.851 | 7 | 0.982 | 8 | 0.897 | 3 | 0.778 | 1.228 | 8 | 6.055 | 7 |
| *TBP* | 0.994 | 9 | 1.079 | 9 | 1.223 | 6 | 0.787 | 1.28 | 9 | 8.132 | 9 |
| *GST* | 0.561 | 1 | 0.465 | 3 | 1.185 | 4 | 0.968 | 0.914 | 3 | 2.449 | 2 |
| *GAPDH* | 0.561 | 1 | 0.671 | 4 | 1.343 | 8 | 0.952 | 1.002 | 4 | 3.364 | 4 |
| *G6PDH* | 0.746 | 6 | 0.695 | 5 | 1.203 | 5 | 0.918 | 1.023 | 6 | 5.477 | 6 |
| *β-TUB1* | 0.613 | 3 | 0.375 | 2 | 1.269 | 7 | 0.991 | 0.88 | 2 | 3.027 | 3 |
| *AK* | 1.078 | 10 | 1.263 | 10 | 1.411 | 9 | 0.846 | 1.415 | 10 | 9.74 | 10 |
| *β-TUB2* | 0.716 | 5 | 0.698 | 6 | 1.442 | 10 | 0.97 | 1.015 | 5 | 6.223 | 8 |
