# Supplementary material for: Reference genes selection for quantitative gene expression studies in tea green leafhoppers, Empoasca onukii Matsuda
Source: PLoS One. 2018 Oct 8;13(10):e0205182. doi: 10.1371/journal.pone.0205182 (PMC6175517; doi:10.1371/journal.pone.0205182)
Supplement: S6 Table — (DOCX) [file pone.0205182.s006.docx]

**S6 Table. Expression Stability of Candidate Reference Genes under All Conditions.**

| **Reference gene** | **RefFinder** | |
| --- | --- | --- |
|  | **Geomean** | **Rank** |
| *GST* | 2.213 | 1 |
| *G6PDH* | 2.3 | 2 |
| *GA* | 3.344 | 3 |
| *β-TUB2* | 3.464 | 4 |
| *UBC* | 4.141 | 5 |
| *α-TUB* | 4.356 | 6 |
| *RPL13* | 5.244 | 7 |
| *TBP* | 6.727 | 8 |
| *AK* | 8.132 | 9 |
| *β-TUB1* | 10 | 10 |
