# Supplementary material for: Reference genes selection for quantitative gene expression studies in tea green leafhoppers, Empoasca onukii Matsuda
Source: PLoS One. 2018 Oct 8;13(10):e0205182. doi: 10.1371/journal.pone.0205182 (PMC6175517; doi:10.1371/journal.pone.0205182)
Supplement: S1 Fig — To assess the robustness of the rankings among the candidate genes, we compared the results obtained using all genes (solid lines), the results obtained when excluding G6PDH (dashed lines) or α-TUB (dot lines) in response to the developmental stages of nymph E. onukii. The results described by geNorm use black curves; red curves represent the values described by NormFinder. The top ranked genes are those with the smallest values for each method. (DOCX) [file pone.0205182.s007.docx]

**S1 Fig. Robustness of geNorm and NormFinder.**
